# *Disinvited, Not Deterred:* College Aspiration and College-Going Conversations Among High School Boys Who Have Been School Disciplined and School Policed

**DOI:** 10.21203/rs.3.rs-10288361/v1

**Published:** 2026-07-14

**Authors:** Collin Perryman, Spencer Platt, Adrian H. Huerta, Nevan Bell

**Affiliations:** The University of Arizona College of Medicine, Tucson, Arizona, U.S.A.; Arizona State University Edson College of Nursing and Health Innovation, Phoenix, Arizona, U.S.A.; Department of Leadership, Learning Design, and Inquiry, College of Education, University of South Carolina, Columbia, South Carolina, U.S.A.; University of Southern California Rossier School of Education, Los Angeles, California, U.S.A.; University of California, Santa Barbara, The Gevirtz School, Department of Education, Santa Barabara, California, U.S.A.

**Keywords:** College aspirations, College-going conversations, Higher educational transitions, School discipline, School policing, Black boys, Latino boys, Latent Class Analysis

## Abstract

**Objective:**

This study identifies college-aspiration and college-going conversation subgroups among 15-year-old high school boys (Black, Latino, White) who have been school disciplined and school policed.

**Methods:**

Data from 1,520 adolescent boys from the Future of Families and Child Wellbeing Study were used to conduct a latent class analysis examining subgroup differences among Black (n = 803), Latino (n = 421), and White (n = 296) boys regarding their experiences with school discipline, school policing, college aspirations, and college-going conversations.

**Results:**

A four-class solution identified four distinct subgroups: (1) Surveilled but Supported, (2) Vicarious Policing, (3) The Criminalized, and (4) The Protected. Covariates included demographics and school connectedness. Auxiliary analyses included multiply imputed R3STEP covariate regression and BCHC sensitivity analysis confirming the primary indicator specification.

**Conclusion:**

Educational carcerality has an adverse impact on the college aspirations and college-going conversations among high school boys, especially Black boys. A multidimensional invitational education framework reveals that carceral school policies function as disinviting forces that undermine the college-going process for boys of color.

A crisis has reached a boiling point for boys and men from Black, Latino/x, Indigenous, Pacific Islanders, and Southeast Asians backgrounds, hereafter, boys and men of color, where nationally, 12% of youth are considered “disconnected,” neither working nor enrolled in school, with Black and Native American youth experiencing the highest rates at 18% and 23% respectively (National Equity Atlas, n.d.). Local and state governments, and pundits are asking, “What’s happening to boys and men?” and “How do we fix this?” and reacting by developing working groups, gender-based scholarships, and enrichment programs to drawback boys and men of color into active members of society (Spaulding et al., 2015; Huerta et al., 2021; Brooms et al., 2018). For the educational pipeline, boys graduate high school on time at lower rates than girls: across the 33 states with available data in 2021, an estimated 81.9 percent of boys graduated on time, compared with 88.4 percent of girls (Reeves & Smith, 2022). These gaps widen sharply by race; in the five large states that report graduation by both sex and race, only 76 percent of Black boys and 78 percent of Hispanic boys graduated on time, compared with 87 percent of White boys and 93 percent of Asian boys (Reeves & Kalkat, 2023). For many people of color, educational attainment is a form of resistance (e.g., Anderson, 1988; Douglass, 1845), and survival to centuries of individual and structural racism, discrimination, which result in negative health outcomes (Assari, 2018a; Assari, 2018b; Jackson et al., 2019; Perryman, 2025, 2026; Perryman et al., 2022; Perryman et al., 2026; Smith, 2004). The case of restricted opportunities is most relevant in the structural limits to educational success for Black, Latino/x, Indigenous, Pacific Islanders, and Southeast Asian boys (George, 2015a), who are often victims of targeted for excessive school discipline practices, detentions, suspensions, and expulsions (Children’s Legal Defense Fund, 1975; Noguera, 2003; Wiley et al., 2024), forms of social control that we term “educational carcerality,” that contribute to school disengagement.

Educational carcerality refers to the importation of criminal justice logic—hyper-surveillance, disproportionate police contact on- and off-school grounds, and punitive discipline—into the learning environment (Shedd, 2015). Recent scholarship increasingly frames the relationship between educational institutions and carceral systems not as a “pipeline” but as a “school-to-prison nexus” (Annamma, 2018; Huerta et al., 2023; Sojoyner, 2016), a framing that captures how schools do not merely feed prisons but increasingly come to resemble them, adopting their technologies, personnel, and logics of control over vulnerable student populations to enforce order over learning.

Though previous research points to unfavorable educational (George, 2015a; Peguero et al., 2015), safety (Gottfredson et al., 2020), and health outcomes (Perryman, 2026a; Perryman et al., 2022) because of pushback from civil rights progress, very seldom does previous research identify and focus on the multi-dimensionalities of educational carcerality as related to college aspirations and college-going conversations among high school boys from a quantitative perspective, using person-centered national-level quantitative analyses. Much of the available empirical scholarship focused on carceral practices than harm college aspirations uses qualitative methods as is limited to singular sites or districts (Carey, 2025/2026; Huerta, 2025; Huerta & Salazar, 2025; Lea et al., 2017; Lea et al., 2022; Rios, 2011).

Furthermore, current research relies on generic measures of “educational aspirations,” which are often assumed to be universally high across racial and ethnic groups (Klasik, 2012). The more consequential metrics, however, are specific college-going conversations—speaking with a counselor, discussing college applications with a teacher, engaging parents or guardians in post-secondary plans, support with financial aid materials, etc.—the tangible actions that constitute the “process” of college access (Huerta, McDonough, & Allen, 2018; McDonough, 1997; Perna, 2006). Our study utilizes data from the *Future of Families & Child Wellbeing Study* (FFCWS) to identify distinct profiles of carceral exposure among Black, Latino, and White boys and to determine how specific combinations of policing and discipline facilitate or stymie college access behaviors.

Because our sample is delimited to boys, we draw on Mutua’s (2013) multidimensionality theory rather than the intersectional frameworks more common in this literature. Multidimensionality extends intersectionality, employing it as part of its own methodology, but was developed specifically to analyze men of color, whose identities sit at the intersection of a privileged gender category and a subordinated racial one, a configuration intersectionality’s dominant applications to women’s lives had not fully modeled. Multidimensionality further accounts for an internal axis of ranking among men themselves, based on perceived masculine performance, that has no clear equivalent in analyses of women’s experiences. This combination of external racial subordination and internal behavioral ranking, what we later term the “privilege paradox,” makes multidimensionality especially suited to theorizing how Black and Latino boys are read and disciplined within schools.

Thus, by utilizing latent class analysis, our study will seek to provide insight into how these carceral mechanisms shape college-going process for Black and Latino/x boys, hereafter, boys of color. Thus, we are guided by the following research questions, such that:

*RQ1:* What is the relationship between educational carcerality and college aspirations among high school aged Black, Latino/x, and White boys?
*RQ2:* What is the relationship between educational carcerality and college-going conversations among high school aged Black, Latino/x, and White boys?

## Variable-Centered vs. Person-Centered Approaches

Existing research on school discipline largely relies on variable-centered, linear approaches such as regression, treating “discipline” and “policing” as independent, additive predictors (see, Skiba, Chung, et al., 2014; Welsh & Little, 2018; Huang & Cornell, 2017). While valuable, this approach is insufficient for understanding how it shapes daily school interaction, the lived experience of carcerality for boys of color. Collins and Lanza (2010) found variable-centered approaches assume population homogeneity. However, carcerality is experienced as a context: a student who attends a school with metal detectors but is never stopped inhabits a fundamentally different climate than one who is frisked daily (Shedd, 2015). A linear model obscures this heterogeneity, failing to distinguish between the student who is simply surveilled and the student who is actively criminalized by educators, school police and other school personnel. By using latent class analysis (LCA), we adopt the person-centered perspective of Collins and Lanza (2010), shifting the focus from variables to individuals and identifying subgroups that experience distinct combinations (Nylund et al., 2007) of educational carcerality and pre-postsecondary experiences.

## Literature Review

### College Aspirations and College-Going Conversations

Having access to a college degree has become increasingly important for many people seeking social mobility (Haveman & Smeeding, 2006). In turn, many high school students are conditioned to have college and university aspirations for reasons such as economic, social, or self-improvement, as college degree and credentials are often the most stable tools for signaling competence and facilitating career opportunities through social capital (Spence, 1973). Many factors influence high school students’ college aspirations or perpetuate barriers (Hill & Wang, 2015; Carey, 2016), depending on several student characteristics that may or may not be within their control. College aspirations are generally influenced by, and associated with, family own college attainment (Agger et al., 2018; Byun et al., 2012; Hill & Wang, 2015), teachers and school counselors (Blanchard & Muller, 2015; Huerta & Lopez, 2025; Jeong Robinson & Roksa, 2016), mental health (Perryman, 2026b; Wang & Peck, 2013), race or ethnicity (Carey, 2016, 2022), carcerality (Huerta, 2025; Huerta & Salazar, 2025), juvenile arrest (Johnson, 2015), and educational policies such as school discipline (Perryman, 2026b; Schollenberger, 2015; Vega et al., 2015).

Familial support is significant in a few ways: adult educational expectations are linked to college aspirations and enrollment (Agger et al., 2018; Perna & Titus, 2005), and students’ college aspirations improve when parental practices such as monitoring, warmth, and autonomy support are present (Hill & Wang, 2015). Families instill educational lessons, values, and beliefs that help students embody postsecondary possible selves (Carey, 2016, 2022). Familial practices matter when high schools’ college-going culture is underdeveloped (McDonough, 1997) or when school counselors’ caseloads preclude individual college advising attention (American School Counselor Association, 2024).

Educators, such as teachers and school counselors, also influence college aspirations, though their influence is mixed. Teachers’ educational expectations are associated with improved college aspirations (Jeong Robinson & Roksa, 2016). On the other hand, teachers’ perceptions can stymie students’ academic progress in their rigorous academic coursework and path towards college preparation (Blanchard & Muller, 2015). Furthermore, educators are often the primary actors who recommend school discipline for students when they violate school norms (Gregory & Mosely, 2004; Skiba et al., 2002), thereby interfering with students’ college aspirations. For example, Schollenberger (2015) used NLSY97 and found that when boys received a school suspension, it resulted in a decrease in bachelor’s degree completion of 28 percentage points among White boys, 12 percentage points among Black boys, and 13 percentage points among Hispanic boys, and this finding has since been confirmed in more recent research which used national data and found that school discipline was associated with less college degree attainment (Perryman, 2026b).

Race and racism play a role in college aspirations as well, since biases against low-income, students of color, children of immigrants, and students enrolled in under-resourced school shape how K-12 educators believe is worthy of the college-going supports Papageorge et al., 2020; Chin et al., 2020). For example, Huerta and Salazar (2025) found in a multisite case study that counselors were reluctant to provide college information to Latino boys who experienced suspensions and expulsions due to believing they were not college material. Because of such structural barriers, communities and families of color are a significant source of social and cultural capital that embolden high school students to pursue their college aspirations and succeed (Delgado, 2023). Delgado (2023) found that older Latina/o siblings often “decoded” the college-going process for younger siblings when high school personnel were unavailable or offered surface-level information about college. When families provide intentional and positive educational messaging to Black and Latino/x high school boys, the boys of color respond by succeeding academically and setting their sights on college (Carey, 2016, 2022).

Finally, and most pertinent to our study, are punitive education discipline policies that confound the students’ academic progress and educational aspirations. In the era of zero-tolerance school discipline policies, students who report lower college aspirations have been suspended for something as small minor as wearing flip-flops to school, talking back to educators, or wearing gang-associated clothing (Huerta et al., 2025; Vega et al., 2015). And more broadly, but still related, research has found that while gang-involved Latino boys have lofty college and career aspirations, the current education system is not set up to serve these boys and other students with similar life experiences, nor do many educational actors wish to avail themselves to help these boys achieve their college aspirations (Huerta, 2025). While the “school-to-prison pipeline” literature is burgeoning across the K-12 educational pipeline (Basile, 2020; Britton & Moreno Luna, 2022; Hirschfield, 2008; Kennedy-Lewis & Mittleman, 2018; Murphy, 2016; Muñiz, 2021; Muñiz & Lizarraga, 2025; Lea et al., 2025, Shedd, 2015), few studies examine how school discipline and policing work together to shape distinct profiles of college aspirations and college-going conversations in a national sample of Black, Latino/x, and White boys. Our study will assist in understanding this by providing knowledge about the relation between educational cacerality (i.e., school discipline and school policing) and college aspirations and college-going conversations among high school aged boys.

### School Discipline and School Policing

School discipline and school policing are well documented to produce unfavorable academic, relational, safety, and health outcomes (Gregory et al., 2010; Skiba, Chung, et al., 2014; Welsh & Little, 2018). Before the 1980s and 1990s, discipline served public relations and non-punitive purposes rather than the standardized, punitive character that would define the zero-tolerance era (Kafka, 2011); in the early 1960s, Southern school boards gained authority to set their own discipline rules, concentrating disciplinary power and setting in motion a new carceral logic in public education (Hale & Livingston, 2023). Recent historiographical work cautions that much of this literature treats the 1990s as the origin point of racialized discipline, when the enabling governance structures were already in motion decades earlier; roughly 60% of studies on Black students and exclusionary discipline invoke history, but overwhelmingly in a narrow, informing capacity restricted to the 1990s, risking a frame that presents the punitive turn as recent rather than a continuation of a longer institutional pattern (Wiley & Middleton, 2025).

That logic hardened with the War on Drugs. The 1986 Anti-Drug Abuse Act allocated $1.7 billion toward drug enforcement, fueling school policing expansion and giving districts wide discretion over disciplinary policy (Nance, 2016). This discretion crystallized into “zero-tolerance,” requiring harsh, predetermined consequences regardless of context, and became codified in state law alongside the 1994 Gun-Free Schools Act (Curran, 2016; Nance, 2016). By 1998, 90% of schools had zero-tolerance policies for firearms, 88% for drugs and alcohol, and 79% for general violence (Forgione, 1998). Though justified initially by gang violence in urban schools of color, the rationale shifted in the late 1990s to protecting suburban, majority-White schools following school shootings, a racialized double standard treated in the literature as defining the era’s uneven application (Addington, 2009; Triplett et al., 2014). Suspension rates for students of color have risen sharply since national data collection began. In 1973, Black students were suspended at 6%, already double the White rate of 3%; by 2010, that gap widened to 16% versus 4% (Leung-Gagné et al., 2022), roughly doubling since the 1970s (George, 2015b; Losen & Gillespie, 2012). The Children’s Defense Fund’s 1975 report remains a foundational touchstone as the first national documentation of these disparities (Wiley & Middleton, 2025); Williams and Wiley (2026) argue the absence of suspension data prior to 1973 has too often been mistaken for absence of the problem itself, rather than a gap in a continuously operating system.

School policing infrastructure grew alongside discipline expansion. From its 1930s origins in Indianapolis, the field has grown to roughly 23,400 sworn officers nationally, 69% of whom report responding to a classroom incident (Davis, 2023). School police presence rose from 10% of schools in 1996–1997 to 64% of high schools by 2014 (Fisher & Devlin, 2019, 2020), 63–64% of middle and high schools in 2013–2014 (Higgins et al., 2020), and 65% by 2019–2020, up from 42.8% in 2009–2010 (Welburn Paige & Bushway, 2024). School police include both sworn and non-sworn personnel (Coon & Travis, 2012); while NASRO frames their mission as threefold, law enforcement, counseling, and educating, officials report their primary function as punitive enforcement despite officers reporting more time spent counseling, a role conflict linked to depressive symptoms among minoritized students (NASRO, 2006; Coon & Travis, 2012; Pamplin et al., 2023). This presence is racially uneven: schools with more Black students are more likely to use zero-tolerance policies, consistent with racial threat theory (Welch & Payne, 2010), and 34–37% of majority-Black or Latinx schools have police present versus 5–11% of majority-White schools (U.S. Department of Education, Office for Civil Rights, 2021). Latinx students face comparable disparities: 1.3 times more likely to be disciplined than White peers nationally (U.S. GAO, 2018), and in Glendale, Arizona, comprising 60% of enrollment but 90% of suspensions (Garcia Mathewson et al., 2022).

Evidence increasingly undermines the premise that punitive discipline responds to differential misbehavior. Race remains a significant predictor of exclusionary discipline even controlling for behavior, SES, and gender (Huang & Cornell, 2017; Skiba, Chung, et al., 2014); disparities stem less from behavior itself than from differential interpretation, with White students referred more for objective infractions and Black students for subjective ones like disrespect or perceived threats (Skiba et al., 2002). School police presence has not consistently improved safety: Theriot (2009) found a 402.3% increase in arrests per 100 students in schools with resource officers, with SES, not officer presence, the strongest predictor of arrest, while zero-tolerance policies account for only about 10% of racial disparities in discipline outcomes and show no consistent safety or behavioral benefit (Curran, 2016; Skiba, 2008). Together, these policies form part of a well-documented pattern of racial disparities (Bottiani et al., 2017; Skiba et al., 2009), unfavorable academic outcomes (Skiba & Rausch, 2004), and adverse health outcomes (Jackson et al., 2019; Perryman, 2025, 2026a; Perryman et al., 2022).

Boys, and boys of color in particular, bear a disproportionate share of this disciplinary and policing burden. Ferguson’s (2000) foundational ethnographic work found that Black boys were adultified within school disciplinary systems, their ordinary childhood misbehavior reinterpreted as willful, threatening conduct deserving of punitive response rather than developmental guidance. This adultification extends beyond the classroom into direct policing contact: Rios (2011) documented how Black and Latino boys are treated as suspects before any infraction occurs, subject to routine surveillance and stops that girls in the same schools and neighborhoods do not experience at comparable rates. Disparities in discipline by family income compound this gendered and racialized targeting, with boys of color facing the steepest cumulative exposure across both economic and racial lines (Barrett et al., 2019). This gendered pattern is not incidental to the broader story of educational carcerality; it signals that the mechanisms driving disproportionate discipline and policing operate specifically on the ways Black and Latino boys’ behavior, presence, and bodies are read within schools, a dynamic that a purely racial analysis would leave only partially explained.

Yet despite this extensive documentation of racial disparity in discipline and policing outcomes, comparatively little research has traced how these carceral experiences shape the college-going process specifically, a gap our study addresses directly. Most existing work on educational carcerality centers academic, safety, or health outcomes in isolation (Gregory et al., 2010; Jackson et al., 2019; Skiba & Rausch, 2004), leaving open how suspension, policing, and vicarious exposure to both combine to shape the conversations and aspirations that constitute the college access process for high school boys. Given that Black and Latinx boys remain disproportionately exposed to both forms of educational carcerality, understanding this relationship is essential not only for documenting harm, but for identifying where in the college-going pipeline that harm actually intervenes.

## Towards a Multidimensional Invitational Education Theoretical Framework

This study integrates two theoretical lenses: invitational education theory (Purkey & Novak, 1996), which frames the school environment, and multidimensionality theory (Mutua, 2013), which frames the gendered and racialized experiences of male students within it. The theory of invitational education helps explain the relationship between school discipline and school policing, and between college aspirations and college-going conversations. Encompassed in this framework are five components: (a) people, (b) places, (c) policies, (d) programs, and (e) processes. For the purposes of our study, we will focus mainly on policies and processes: policies refer to school discipline and school policing as “disinviting” forces, and processes refer to college-going conversations such as students seeking counseling and mentorship. We posit that disinviting carceral policies undermine the inviting processes necessary for college access (Vega et al., 2015).

Further, this study focuses exclusively on males to address gendered racism through Mutua’s (2013) multidimensionality theory. Mutua suggests that multidimensionality theory understands the nexus of arduous gendered racism against male of color targets, and further states that males of color comprise of internal dimensions that exhibit multiple rankings, levels, and degrees of behavioral performance, and external dimensions that allocate men of color into hierarchized levels of race and class. This complexity of internal and external dimensions satisfies what we refer to as the “privilege paradox”—the tension of occupying a dominant gender category (male) while navigating a subordinate racial category. Black and Latino boys face a unique “adultification bias” where their behaviors are misinterpreted as threatening at younger ages (Ferguson, 2000; Rios, 2011), leading to harsher penalties (Barrett et al., 2019). While Black girls also face disproportionate discipline (Annamma, 2018), the mechanisms operate through different theoretical pathways, as Mutua distinguishes multidimensionality from intersectionality by highlighting how male privilege interacts with, and is constrained by, racial oppression.

Taken together, a multidimensional theory of invitational education guides our thinking about how the enactment of educational policies, such as school policing and school discipline, relates to the college aspirations and college-going conversations of 15-year-old high school boys. We argue that carceral policies are not neutrally applied but are activated by the specific external dimensions of race, gender, and class of male students. Zero-tolerance policies function as targeted disinviting responses to the gendered-racial performance of Black and Latino boys, with the school environment reading their multidimensional identity not as potential to be nurtured and encouraged, but as risk to be managed, thereby severing the processes of college access.

## Purpose and Hypotheses

Therefore, we hypothesized that there would be nuanced subgroup differences among high school-aged Black male students, Latino male students, and White male students in terms of their college aspirations and college-going conversation, defined by their experiences with (1) ever being suspended or expelled in the past two years, (2) police officer or officers regularly stationed at school, (3) some other security guard regularly stationed at school, (4) being stopped by police at school, and (5) ever seeing someone stopped by police in their school.

## Methods

### Study Population

The current study used data from the Year 15 wave of the Future of Families and Child Wellbeing Study (FFCWS). The FFCWS is a nationally representative birth cohort study that surveyed approximately 4,700 births across 75 hospitals in 20 large U.S. cities between 1998 and 2000 (Reichman et al., 2001). The sampling design oversampled non-marital births and minority families, making the dataset uniquely suited for examining carcerality among populations often underrepresented in general surveys. The Year 15 wave captures focal children at approximately age 15—a critical developmental window in which the transition to high school coincides with intensified contact with both the criminal justice system and the colleg-preparation pipeline. After delimiting the sample to Black (n = 803), Latino (n = 421), and White (n = 296) boys, our final analytic sample was N = 1,520.

### Distal Categorical Outcomes

College aspiration and college-going conversations are categorical indicators were treated as distal categorical outcomes in the BCHC sensitivity analysis. College aspirations included the following questions: (a) “How important to you that you graduate from high school?”; (b) “How important to you that you graduate from college?” and (c) “How likely are you to graduate from college?” College-going conversations included the following questions: (d) “Have you talked to an adult about applying to college?”; (e) “Talked to parent/guardian about college”; (f) “Talked to guidance counselor about college”; and (g) “Talked to teacher/coach about college.” Talking to a parent or guardian, talking to a guidance counselor, and talking to a teacher or coach were asked only of respondents who indicated having talked to an adult about applying to college; respondents who answered no to this gate item skipped the three follow-up items by design. Analyses of these three indicators are therefore based on n = 999, conditional on talking to an adult = 1, and should be interpreted as comparisons among respondents who had already engaged in some form of adult conversation about college rather than as population-wide estimates.

In the BCHC specification these variables were removed from the class-defining indicator set and estimated as BCHC-corrected distal categorical outcomes, allowing assessment of whether aspirations and conversations differed significantly across classes defined solely by carceral exposure. Because these indicators are categorical, the categorical BCH extension (BCHC) was used rather than the continuous BCH specification, since BCHC tests equality of category probabilities across classes and provides class-comparison odds ratios rather than mean differences (Asparouhov & Muthén, 2021).

### Latent Variable Indicators

School discipline and policing were yes/no indicators and included: (a) “Ever been suspended or expelled in past 2 years?”; (b) “Police officer or officers regularly stationed at school?”; (c) “Some other security guard regularly stationed at school?”; (d) “Stopped by police at school”; and (e) “Ever seen someone stopped by police in your school?” Race/ethnicity was categorized as Black, Hispanic/Latino, and White and were included as a class indicator rather than a covariate, consistent with the theoretical argument that racial position is constitutive of carceral exposure rather than a predictor of it.

### Covariates

Demographic covariates included these variables: maternal marital status (married vs. not married), maternal education level (less than high school, high school or equivalent, some college or higher), maternal income (up to $14,999, $15,000 to $34,999, and $35,000+), and maternal nativity (born in the United States or elsewhere). School-level covariates included these variables: “I feel like I am part of my school”; “I feel close to people at my school”; “I am happy to be at my school”; “I feel safe at my school”; and “Teachers in school treat the student with respect.”

### Missing Data

Covariance coverage across the 13 latent class indicators ranged from 0.366 to 1.000. The stopped by police at school indicator had the lowest covariance coverage at 36.6%, reflecting meaningful item-level missingness on this variable. All other indicators exceeded 95% of coverage. Missing data on auxiliary covariates were addressed through Bayesian multiple imputation, generating 20 imputed datasets in Mplus Version 9. The imputation model included all latent class indicators and auxiliary covariates. Pooled R3STEP results across 20 imputed datasets are reported alongside listwise results for transparency.

### Analytic Strategy

The current study first examined sample characteristics of the respondents. Second, the study underwent a latent class analysis (LCA) following Collins and Lanza (2010) to identify college aspirations and college-going conversation profiles of respondents about the indicators of school discipline and school policing. A comparative approach was taken to choose the best LCA model for interpretation, where one to four class solutions were compared. Criteria for model selection included the Akaike information criterion (AIC), Bayesian information criterion (BIC), sample-size adjusted BIC (aBIC), entropy, the Lo-Mendell-Rubin likelihood ratio test (LMR-LRT), and the parametric bootstrapped likelihood ratio test (BLRT). The four-class solution was selected based on the lowest AIC (17466.16) and sample-size adjusted BIC (aBIC = 17593.00), a significant BLRT (p < .0001) and LMR-LRT (p = .0001), and superior substantive interpretability. Raw BIC was nearly identical between the three- and four-class solutions (17779.21 vs. 17780.42, a negligible difference) and entropy was equivalent (0.67 for both), so model selection was guided by theoretical coherence: the three-class solution collapsed the vicarious policing and direct discipline subgroups into a single undivided class, obscuring a theoretically meaningful distinction that the four-class solution preserves. The five-class solution was rejected because the LMR-LRT was nonsignificant (p = .12), BIC increased, and entropy declined to 0.61. Average latent class posterior probabilities (AvePP) for the four-class solution ranged from 0.787 to 0.936, meeting the threshold of 0.70 recommended for acceptable classification quality (Nagin, 2005). The BLRT for the four-class solution was based on five successful bootstrap draws due to computational constraints; the significant LMR-LRT (p = .0001) independently corroborates class enumeration. One threshold parameter was fixed during estimation due to near-singularity in Class 2.

To examine local independence, TECH10 bivariate residuals were inspected. Standardized residuals indicated violations concentrated among college-going conversations indicator pairs and among the two police contact indicators (stopped by police at school and ever seen someone stopped by police in your school), consistent with shared method variance. These violations are theoretically expected given the co-occurring nature of college-going conversations and the structural co-occurrence of school police and security personnel, and do not indicate arbitrary model misfit.

Auxiliary analyses were conducted following Asparouhov and Muthén (2014) using the three-step R3STEP procedure to examine covariate associations with class membership while preserving the class solution. To address missing data on auxiliary covariates, 20 multiply imputed datasets were generated using Bayesian multiple imputation via Mplus, and the R3STEP procedure was conducted across all 20 datasets with pooled results. The listwise auxiliary regression (n = 1,137) and the multiply imputed regression (N = 1,520) produced substantively identical findings, confirming that missing data did not bias covariate results.

A BCHC sensitivity analysis (Bolck et al., 2004; Vermunt, 2010; Asparouhov & Muthén, 2021) was conducted to evaluate whether college aspiration and college-going conversation indicators were constitutive of the class structure or downstream outcomes. In the BCHC specification, the class solution was estimated using only the carceral indicators, with aspiration and conversation variables treated as distal categorical outcomes. The resulting BCHC classes showed substantially lower entropy (0.53 vs. 0.67). Overall equality tests across classes were nonsignificant for six of the seven distal outcomes (H”ow important to you that you graduate from college?”; “How likely are you to graduate from college?”; “Have you talked to an adult about applying to college?”; “Talked to parent/guardian about college”; “Talked to guidance counselor about college”; “Talked to teacher/coach about college”; all p > .09), while “How important to you that you graduate from high school?” differed significantly across classes (p < .001), driven primarily by Class 2, in which the item was answered affirmatively by all respondents. This pattern suggests that most college aspiration and college-going conversation indicators are jointly constitutive of the carceral class structure rather than separable downstream outcomes, with the exception of high school graduation importance, which functions somewhat independently of class assignment. Race was included as a class indicator rather than a covariate, consistent with the theoretical argument that racial position is constitutive of carceral exposure rather than a predictor of it. All analyses were conducted in Mplus Version 9.

## Results

### Sample Characteristics

Table 1 presents sample characteristics of the respondents. Black boys reported the highest suspension rates (40.9%) compared to Latino (26.3%) and White boys (16.0%), and security guard presence was higher in schools attended by Black boys (78.2%) compared to White boys (52.6%). Despite these disparities in discipline and policing, college aspirations remained universally high across all racial groups, with over 97% reporting that college was important—underscoring the critical distinction between aspirations and the college-going conversations that our study seeks to illuminate.

### Latent Class Model

Table 2 presents a four-class model solution that was selected based on model fit criteria. Classification quality was acceptable, with average latent class posterior probabilities (AvePP) ranging from 0.787 to 0.936 across the four classes, all exceeding the recommended threshold of 0.70 (Nagin, 2005). Using an interpretive approach, the subgroup profiles were labeled for respondent reports of college aspirations, college-going conversations, school discipline, and school policing.

Table 3 presents Class 1 (43% of the sample), which was categorized as “Surveilled but Supported.” The educational aspirations of the boys in this profile were: these boys responded that it was important to them that they graduate from high school (100%), it was important to them that they graduate from college (100%), and it was likely they are to graduate from college (100%). Most of this profile had a police officer or officers regularly stationed at school (84%), along with some other security guard regularly stationed at school (83%). About 45% of this group had ever been suspended or expelled in the past two years, and 23% had been stopped by police at school. In spite of heavy school police presence and moderate vicarious school policing, most of these boys (69%) talked to a teacher or coach about college, while about half (51%) talked to their parents or guardians. Most of this profile were comprise of Black (71%) and Latino (29%) boys. No White boys were in this class—highlighting that educational carcerality is exclusively experienced by boys of color in our national sample.

Table 3 presents Class 2 (29.7% of the sample), which was categorized as “Vicarious Policing.” The educational aspirations of the boys in this profile were: these boys responded that it was important to them that they graduate from high school (100%), it was important to them that they graduate from college (100%), and it was likely they are to graduate from college (100%). Most of these boys had a police officer or officers regularly stationed at school (86%), along with some other security guard regularly stationed at school (80%). Just over half of these boys (58%) ever saw someone stopped by police in their school. All these boys reported educational aspirations, and all had spoken with an adult about applying to college. All of these boys reported talking to an adult about college. Specifically, 31% talked to a teacher or coach and 38% talked to a parent or guardian about college. By contrast, 100% of these boys talked to a guidance counselor about college. Most of this profile were comprised of Black (59%), Latino (27%), and White (15%) boys.

Table 3 presents Class 3 (4% of the sample), which was categorized as “The Criminalized.” This was the smallest but perhaps most critical group. Almost all of these boys thought it was important that they graduate from high school (96%). However, just over half of these boys thought it was important to them that they graduate from college (57%) and that it was likely they were to graduate from college (51%). This was the only class where college aspirations declined markedly: the probability of viewing college as important dropped to 57%, and the probability of viewing college as “likely” dropped to 51% (vs. 98–100% in other classes). These boys had the highest conditional probabilities in the entire sample of having a police officer or officers regularly stationed at school (97%), ever being suspended or expelled in the past two years (52%), and being stopped by police at school (38%). The boys in profile had the lowest probability in the entire sample of talking to any adult about college (30%), indicating a severe severance of college-going conversational ties to adults. Of the adults they did talk to about college hovered around a similar probability: parents or guardians (26%), guidance counselors (39%), and teachers or coaches (35%). Most of this profile were Black (57%), Latino (21%), and White (23%) boys. (Conditional probabilities should be interpreted with caution given the small cell size.)

Finally, Table 3 presents Class 4 (23.4% of the sample), which was categorized as “The Protected.” Most of this profile had not been suspended or expelled in the past two years, with only 13% reporting suspension, and they had the lowest police stop conditional probability (16%) and lowest witness probability (38%). Almost all boys reported their educational aspirations, and most talked to an adult about applying to college; specifically, 71% talked to a parent or guardian. Most of this profile were White boysq (50%), then Latino boys (28%) and Black boys (23%) male students. The presence of Latino boys here is noteworthy, and discussion of bifurcation is below.

**Figure 1.**
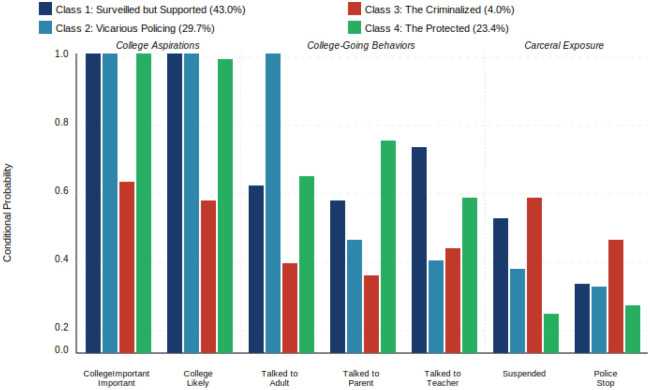
Conditional Probability Profiles for the Four-Class Solution *Note*. Bars represent the conditional probability of each indicator response given class membership. Indicators are grouped by domain: college aspirations (college important, college likely), college-going behaviors (talked to adult, parent/guardian, teacher/coach), and carceral exposure (suspended past two years, stopped by police). Class 3 (The Criminalized, n = 61) is the only class showing markedly reduced college aspiration and college-going conversation probabilities.

Figure 1 presents the conditional probability profiles for all four classes across the college aspiration, college-going conversation, and educational carcerality indicators, illustrating the results above.

### Membership Analysis: Auxiliary Regression Results

As presented in Table 4, maternal marital status was the strongest and most consistent predictor of class membership across all specifications. In the multiply imputed auxiliary regression (N = 1,520), boys with married mothers were significantly less likely to be in Class 1 (OR = 0.055, 95% CI [0.020, 0.152]), Class 2 (OR = 0.255, 95% CI [0.164, 0.398]), or Class 3 (OR = 0.260, 95% CI [0.113, 0.597]) compared to the Protected class. These estimates were stable across the listwise (n = 1,137) and multiply imputed specifications, with MI estimates modestly stronger in magnitude, consistent with the expectation that listwise deletion disproportionately excluded cases with missing covariate data. No other demographic or school connectedness covariate reached significance consistently across classes. Among school connectedness variables, happiness at school was associated with reduced odds of membership in the Criminalized class (Class 3) in the listwise specification (OR = 0.27, 95% CI [0.08, 0.89]); this association did not reach significance in the multiply imputed model, suggesting it should be interpreted with caution.

## Discussion

The purpose of our study was to understand the nuanced subgroups in college aspirations, college-going conversations, school discipline, and school policing experienced among high school boys, using a national study. Therefore, we asked whether educational carcerality influenced the college aspirations and college-going conversations with adults among these boys. To this end, we found a few key findings that will be discussed.

### Perseverance Despite Educational Carcerality

Despite the persistence of educational carcerality disproportionately experienced by Black boys in our study (Classes 1–3), a majority of these boys actively maintained aspirations to graduate from college while taking concrete steps toward that goal, whether by talking with parents, teachers, or counselors about applying. Even in Class 1, “Surveilled but Supported,” that was exclusively comprised of boys of color (71% Black and 29% Latino) experienced policing exposure, yet all these boys had educational aspirations and nearly 70% spoke with a teacher or coach about college. This pattern held even in Class 2, “Vicarious Policing, where more than half were boys of color (59% Black and 27% Latino) had experienced vicarious policing exposure (i,e., seeing another student being stopped), and yet every one of these boys had spoken with an adult about applying to college, and of these that spoke with an adult 100% of them spoke with a counselor. Only the boys Class 3, “The Criminalized,” the smallest and most carcerally exposed class and comparable racial composition as Class 2, did college aspirations meaningfully decline (i.e., 57%: graduating from college was important; 51%: graduating from college was likely)--suggesting that the erosion of college-going beliefs and conversations is not a general condition of surveillance but a response specific to the most concentrated and direct forms of criminalization.

These findings push back against long-held, persistent, and deficit-based narratives that have frame boys of color as disengaged from or indifferent to educational attainment (Carey, 2016, 2022; Ferguson, 2000). Instead, our results reposition these boys as optimistic, active agents who continue pursuing both high school and college graduation even while navigating carceral exposures in their own schools. This perseverance does not minimize the harm educational carcerality inflicts; rather, it clarifies where that harm actually lands. The damage carceral schooling does is not primarily to boys’ belief in the value of education, but to the social infrastructure, namely their access to trusted adults and college-going conversations, that would otherwise translate that belief into action. Recognizing this distinction matters for practice and policy alike: interventions aimed at restoring hope among boys of color misdiagnose the problem, while interventions aimed at rebuilding the relationships and support systems severed by criminalizing address the mechanism our findings identify.

### Fractured College-Going Conversations due to Educational Carcerality

For the Black and Latino boys who comprised Class 1 (“Surveilled but Supported), carcerality—i.e., police and security regularly stationed at school—did not diminish their educational aspirations, highlighting the inequities related to regularly stationing of police and security on high school campuses. Across all classes, the predominant indicator influencing college-going conversations were school discipline and being stopped by school police, especially for boys of color. Boys of color who mostly comprised Class 3 (in the ‘Criminalized’ class (Class 3) heavily experienced educational carcerality and were least likely to speak with an adult or a parent, though their probability of speaking with a counselor about college was comparable to or higher than most other classes, and their probability of speaking with an teacher was low (35%). Both school discipline and school policing served as a deterrent to college-going conversations of Class 3, which is overwhelmingly composed of boys of color. Similarly, boys in Class 2 vicariously saw other students being stopped by school police were less likely to speak with their parents and teachers about going to college. For White boys who mostly comprised Class 4 (“The Protected”) were suspended and expelled the least in the entire sample, and they reported a low probability of speaking with their teachers and counselors within this class. Considering that White boys comprise most of the “Protected” class, this finding raises the questions as to why they have less college-going conversations than their peers, whose lack of college-going conversations explained in part by educational carcerality.

Inherently, this implicates adult supporters into the school-to-prison nexus, implying that if students are not having conversations around college as a product of educational carcerality, that adults are not helping mitigate this clear inequity. If White boys are reporting less college conversations compared to their peers of color, then what boys is being offered help about college? How can any community of boys engage in college-going conversations if this support is in relation to the policies and processes that are interwoven with school discipline and school policing?

### Vicarious Policing and Its Consequences

For the boys of color who are mostly comprised of Class 2 (“Vicarious Policing”) highlights a subtle but pervasive impact of educational carcerality: vicarious policing. Though not directly experiencing policing exposure, boys in this profile witnessed the impact of their peers being stopped by school police stops. Consequentially decreasing college-going conversations with teachers below that of Class 1, the results reveal vicarious policing may undermine these conversations in the college preparation process. Further, educational carcerality on criminalized the boys of color in Class 3, held an indirect adverse outcome on these boys who witnessed policing on other students. Severing the connections to adults, thus, negatively impacts their ability to have college-going conversations for boys primarily and secondarily impacted by school policing.

Nationally, the Civil Rights Data Collection (CRDC) Office for Civil Rights tracks suspensions and arrests through ecological-, distal-level but that does not capture vicarious school policing activities. FFCWS data, however, is had this opportunity, because it captures individual-level data of children and adolescents like the boys in our study. Invisible to existing federal accountability mechanisms, these boys are then not reported in any federal dataset as experiencing harm from educational carcerality and the like. At a moment when the federal infrastructure for civil rights enforcement is being restructured and revaluated, our findings reveal the urgent need for instrument development at the ecological level that expands upon measurements of carceral schooling.

### “The Criminalized” Class and the Undermining of Process

For the boys of color who mostly comprise Class 3 (“The Criminalized”), experienced both the combination of school discipline and directly being stopped by police at school. The experiences that our study found about these boys provided empirical support for the school-to-prison nexus. Boys in this class reported disengaging from college preparatory processes. As the only group where college aspirations declined, this finding supports the argument that educational carcerality function as a “disinviting” force (Vega et al, 2015). Affirming our theoretical framework of multidimensional invitational education to understand the influence of educational carcerality on college-going conversations of high school boys of color, these exposures of school discipline policies encouraging criminalization, such disproportionate school discipline and policing that undermines the college-going conversations that are essential in successful college-going processes.

### The Bifurcation of Latino Boys

Our LCA reveals what we describe as a bifurcated reality for Latino boys: they comprised 29% of Class 1 (Surveilled abut Supported) and 27% of Class 2 (Vicarious Policing), sharing heavily policed environments with their Black peers, yet they also comprised 28% of the Protected class. This finding suggests that a subset of Latino boys—perhaps through colorism, geography, or assimilation trajectories—may navigate the educational system in ways that more closely resemble the experience of White boys, while other Latino boys experience school discipline and policing alongside Black peers. This bifurcation is important because it disrupts monolithic narratives about Latino experiences in schools and suggests that interventions must target the specific environments where Latino boys are surveilled while also acknowledging that other Latino boys face different barriers or have different experiences.

### Previous Empirical Research and Directions for Future Research

Our findings corroborate and extend a body of qualitative scholarship documenting how educational carcerality erodes the relationships that sustain boys’ college trajectories. Huerta’s (2022, 2025) case studies of gang-associated and gang-involved Latino boys found that educators frequently withheld college knowledge from students they perceived as unworthy of investment, treating disciplinary history as evidence of diminished college potential rather than as a structural harm requiring redress, a pattern our results substantiate at scale: boys in Class 3 (“The Criminalized”) reported the lowest probability of talking to any adult about college despite a probability of speaking with a counselor comparable to or higher than most other classes, suggesting the withholding Huerta describes operates selectively even where formal advising channels remain nominally available. Huerta et al. (2023) similarly found that criminalized Latino boys possessed genuine college aspirations that rarely translated into actionable college knowledge, consistent with our finding that college aspirations remained near-universal across most classes even as college-going conversations diverged sharply by carceral exposure. Carey’s (2016, 2022) qualitative work on Black and Latino boys’ college-going processes found that family messaging could partially buffer against school-based disinvestment, though Carey (2025) later documented how criminalization within charter settings actively undermined these protective factors, a pattern our auxiliary regression results echo: maternal marital status was the strongest and most consistent predictor of class membership across all specifications, suggesting family-level resources continue to shape carceral exposure and its consequences even within a national sample. Rios’s (2011) ethnographic account of the “youth control complex” described how punitive school responses instilled a sense of foreclosed futures among Black and Latino boys well before any contact with the criminal legal system, language that resonates with our finding that Class 3, defined by the highest concentration of both discipline and policing exposure, was the only class where college aspirations meaningfully declined. Where these qualitative studies illuminate the mechanisms of disinvestment through rich, situated accounts within single schools, districts, or populations, our study demonstrates that the pathway they describe is statistically detectable across a national sample of Black, Latino, and White boys, lending quantitative agreement to what qualitative scholars have long argued.

Our findings also extend and refine prior quantitative research linking educational carcerality to diminished educational outcomes. Shollenberger’s (2015) NLSY97 analysis established that suspension predicts substantially reduced odds of high school completion, college attendance, and bachelor’s degree attainment, finding a 28 percentage-point decrease in degree completion among suspended White boys, 12 points among suspended Black boys, and 13 points among suspended Hispanic boys; our study extends this work by demonstrating that discipline’s consequences are not uniform even within racial groups, but instead depend on the specific configuration of discipline and policing exposures, with only the most heavily surveilled and directly policed subgroup (Class 3) showing measurable declines in college aspirations. Using GSEM mediation analysis on this same dataset, Perryman (2026b) found that school discipline significantly predicted higher depressive symptoms (b = 0.46, p = 0.001), that discipline showed no significant direct effect on college-going behaviors (b = −0.08, p = 0.090) once depressive symptoms were accounted for, and that discipline retained a significant direct effect on degree attainment (OR = 0.41, p = 0.010) independent of the depressive pathway; our person-centered findings complement this variable-centered account by showing that the psychological and behavioral consequences Perryman (2026b) traces through mediation are themselves unevenly distributed, concentrated most heavily among boys of color in the Criminalized and Vicarious Policing classes rather than uniformly across all disciplined students. Skiba et al. (2011) and Skiba, Chung, et al. (2014) similarly used large administrative datasets to document racial disparities in discipline exposure and downstream achievement gaps using variable-centered regression approaches; our findings corroborate their core conclusion that race remains a significant predictor of exclusionary discipline independent of behavior, while demonstrating that a person-centered approach reveals meaningful within-race heterogeneity, particularly the bifurcation of Latino boys across both heavily policed and protected profiles, that variable-centered models are not less likely to detect.

Taken together, the qualitative and quantitative literatures reviewed above a converging story from different vantage points. Educators withhold college knowledge from disciplined students (Huerta, 2022, 2025; Huerta et al., 2023), families buffer against but cannot fully offset this disinvestment (Carey, 2016, 2022, 2025), and punitive contact forecloses a sense of educational futurity well before criminal legal involvement (Rios, 2011). These dynamics are statistically detectable at a national level through both variable-centered mediation (Perryman, 2026b) and the person-centered profiles identified in our current study. Yet each tradition also reveals a gap the other cannot close on its own. Qualitative work explains the mechanisms of disinvestment in rich, situated detail but cannot establish how widely those mechanisms reach or which students are most affected. Variable-centered quantitative work (Perryman’s, 2026b; Shollenberger’s, 2015) can establish that relationships exist at a population level but, by design, averages across the very heterogeneity our four-class solution reveals, obscuring the fact that the Criminalized and Vicarious Policing profiles bear this burden very differently than the Surveilled but Supported or Protected profiles do. Person-centered work like the present study bridges this gap empirically, but does not, on its own, explain why particular boys land in particular classes or how they narrate that experience in their own words.

Future research should therefore move in three complementary directions. First, mixed-methods designs that pair person-centered latent class or profile analyses with in-depth interviews of boys within each identified class would allow researchers to test whether the mechanisms qualitative scholars have documented in single sites, such as counselor withholding or familial buffering, operate differently across the Surveilled but Supported, Vicarious Policing, Criminalized, and Protected profiles identified here, rather than assuming uniform application across all disciplined students. Second, longitudinal quantitative and qualitative research is needed to establish temporal ordering and test causal pathways that the cross-sectional and single-wave designs common in this literature, including the present study, cannot support; tracking these same class profiles across multiple waves would clarify whether class membership itself predicts downstream degree attainment, extending Perryman’s (2026b) mediation findings into a person-centered longitudinal framework. Third, historiographical work remains underutilized in this literature despite its capacity to correct the ahistorical framing that treats racialized school discipline as a contemporary or 1990s-originating phenomenon (Wiley & Middleton, 2025); future mixed-methods, qualitative, and quantitative studies would benefit from explicitly situating their work within the longer institutional histories of school-based racial control, rather than treating each new dataset as evidence of a novel problem. No single method can capture the full architecture of educational carcerality’s relationship to college access; the path forward lies in triangulating mechanism, distribution, temporality, and history together.

### Theoretical Synthesis: Disinvitation and the Privilege Paradox

Our findings substantiate the utility of pairing invitational education theory with multidimensionality theory to understand how educational carcerality operates on boys of color. Invitational education theory frames school discipline and school policing as disinviting policies that should, in theory, suppress the inviting processes necessary for college access, namely the conversations with parents and guardians, teachers and coaches, and counselors that translate aspiration into action (Purkey & Novak, 1996; Vega et al., 2015). Our four-class solution confirms this relationship, but only under specific conditions. Boys of color who largely comprised Class 3, “The Criminalized,” experienced the highest concentrations of both discipline and direct police contact, and this class alone showed a marked decline in college aspirations alongside the lowest probability of speaking with any adult about college. This pattern is precisely what invitational theory would predict when disinviting forces reach a critical threshold: the erosion of both the inviting processes and the underlying belief those processes are meant to support. Yet Classes 1 and 2, despite substantial exposure to discipline and policing, retained high educational aspirations even as their college-going conversations narrowed. This divergence suggests that disinviting policies do not uniformly disinvite; rather, their capacity to sever the college-going process depends on the intensity and directness of disinviting carceral contact—a nuance that a purely ecological policy-level application of invitational theory would not have captured without our person-centered approach using individual-level data.

Multidimensionality theory clarifies why this carceral exposure and its consequences were so unevenly distributed by race within the four classes. Consistent with the “privilege paradox,” Black and Latino boys occupied the overwhelming majority of Classes 1 through 3, while White boys were concentrated in Class 4, “The Protected,” and entirely absent from Class 1. This stratification illustrates Mutua’s (2013) argument that boys of color are read not through the privileged gender category they nominally occupy, but through the external racial hierarchy that subordinates them, rendering their multidimensional identity legible to school actors primarily as risk rather than potential. The bifurcation of Latino boys across both heavily policed and protected classes further demonstrates multidimensionality’s attentiveness to internal variation within a racial group, complicating any assumption that Latino identity confers a uniform carceral exposure. Taken together, our findings suggest that educational carcerality functions multidimensionally: it is activated by external racial positioning, moderated by the intensity of direct versus vicarious contact, and unevenly disinviting depending on where a given subgroup of boys falls along these various axes, a complexity that neither theory alone would have fully revealed.

### Limitations and Strengths

Though our study is novel, and highlights the experiences of boys of color, and employs nuanced methods of a national sample, there are some points that bring pause in our study. First, our study uses one wave of data, and therefore, longitudinal or causal conclusions cannot be drawn. Second, the school discipline and school policing variables are binary, providing only two experiences for each question, which may mask frequency, severity, or dose-response pertaining to the impact of educational carcerality. Third, our study effectively demonstrated that boys who experience educational carcerality and college-going conversation;s however, our measures of educational carcerality (school discipline and school policing) are not the sole punitive encounters during their educational journeys and do not exclusively define the educational experiences of the boys of color in our study. Third, Class 3 (n ≈ 61) warrants cautious interpretation given the small cell size, though the Parametric Bootstrapped Likelihood Ratio Test (BLRT), as found in the Methods section, supported its inclusion.

Notwithstanding the points of pause, our study reached new heights and substantially moves the literature forward in advocating for the civil rights of high school boys, and high school Black boys in particular. First, while there are studies on the carceral and higher educational aspirations among high school boys, few are quantitative and reveal national results. Second, not only did our study use national data, but it employs latent, person-centered modeling (i.e., LCA), which provides both the nuance of individual respondents, while also allowing for national results and findings. Third, our study uses a sample with a significant number of boys from minoritized communities, which is rare in nationally representative studies, thus, allowing our study to center their nuanced and rarely discussed experiences. Fourth, our study delimited an analytic sample of exclusively boys, which is important because it allowed us to understand within-gender experiences, differences, and similarities that could be overlooked otherwise. Fifth, our study innovates a theoretical synthesis of multidimensionality and invitational education and theories, providing a framework for understanding how educational policies interact with the nuanced, gendered-racial identities experienced by boys of color.

### Conclusion: Implications for Policy and Practice

We conclude by stating that the person-centered approach of LCA enabled precise policy and practice recommendations that attend to the nuanced experiences of the boys of color identified in our study. We, therefore, offer nuanced recommendations across three areas within the educational enterprise: high schools, higher education, and educational transitions between the two.

### High Schools

As federal civil rights enforcement is restructured, state- and district-level mandates become critical for making visible the patterns our study documented. Drawing on invitational education theory, we call on districts to establish binding memoranda of understanding abolishing school police involvement in non-violent code of conduct violations, interactions disproportionately levied against boys of color that also carried negative effects for boys who merely witnessed them. State Departments of Education should require police stop data disaggregated by race and gender, transparency that may reveal the multidimensional gendered and racialized experiences our analysis captured. High schools should also extend these findings into training for counselors and other student support staff, with school psychologists recognizing witnessing peer criminalization as a form of secondary trauma and activating trauma-informed care protocols for bystanders, not only students directly disciplined or stopped by police. Calling on Carey (2025) alongside our own findings on disproportionate policing and discipline, we posit that educational systems benefit from destigmatizing and decriminalizing disciplinary and structural power, work that requires transforming both district policy and the underlying school culture that sustains these harms; we urge schools to center the multidimensional experiences of boys of color, foster proactive conversations between supporters and students about college attendance, and work to build aspiration, safety, and belonging rather than reproducing the oppressive mechanisms that marginalize students elsewhere in society.

### Higher Education

Guided by the goal of encouraging student college-going conversations and educational aspirations, universities must consider the implications of criminalization for boys of color throughout the college admissions process; recognizing that these harms have already come to pass, institutions have all the more reason to name these inequities for boys who have been both directly and indirectly impacted by carceral systems embedded in their educational journeys, particularly given that harmful practices are often reproduced through institutional isomorphism and rationality (DiMaggio & Powell, 1983) rather than through leadership actively innovating with these students’ experiences in mind. Higher education institutions may instead enact contextualized admissions reviews that view discipline records through a restorative, structurally-informed lens, leveraging their unique positionality to rebuild trust eroded by carcerally punitive discipline and policing; universities should eliminate discipline questions from applications and deploy external college access professionals in high-surveillance schools, bypassing school-level infrastructure where that trust has been demonstrated to be compromised.

### Educational Transitions

This study highlights age 15 as a pivotal moment in the college-going process alongside intensifying contact with the criminal justice system; schools should implement transition programs for Black and Latino boys that buffer against the weight of high school surveillance at this milestone, bolstering the inviting processes necessary to sustain college-going conversations within carceral educational environments, an approach that has proven successful in encouraging educational aspirations and persistence in university settings (Huerta et al., 2021; Brooms et al., 2018) and may be intentionally adapted for high school boys of color. The bifurcation of Latino boys observed in our findings also complicates the monolithic narratives that have long followed boys of color through the transition from adolescence in high school into adulthood in college; given the structures of colorism and white supremacy that shape this variation, educators and supporters alike should attend to both the multidimensional expressions of identity and the range of student narratives as boys move through these educational milestones.

At a moment when the federal infrastructure for protecting students’ civil rights is being restructured, the findings underscore precisely why that infrastructure matters. The four latent classes—and the racial stratification defining them—are visible only because nationally representative, disaggregated data exist. Ultimately, the fight for the educational rights of boys of color is a continuation of a centuries-old struggle. Just as Frederick Douglass once pursued literacy in the face of brutal opposition, today’s Black and Latino boys are pursuing a future in the face of surveillance. Therefore, it is incumbent on all of us to ensure that the modern schoolhouse honors this legacy—not as a site of containment and control, but as the true “pathway from slavery to freedom” that Douglass envisioned, where literacy is finally realized as liberation for all boys of color.

## Figures and Tables

**Table 1.** Sample Characteristics by Race/Ethnicity, Future of Families and Child Wellbeing Study Year 15 (N = 1,520)

Variable	Total Sample (N = 1,520)	Black Boys (n = 803)	Latino Boys (n = 421)	White Boys (n = 296)
*Educational Aspirations*				
How important to you that you graduate from high school? (Important)	99.7%	99.9%	99.0%	100.0%
How important to you that you graduate from college? (Important)	97.6%	97.1%	98.8%	97.3%
How likely are you to graduate from college? (Likely)	96.6%	97.0%	96.9%	94.9%
*College-Going Conversations*				
Have you talked to an adult about applying to college? (Yes)	65.8%	68.2%	61.9%	64.9%
Talked to parent/guardian about college (Yes)^a^	51.1%	49.5%	44.2%	64.6%
Talked to guidance counselor about college (Yes)^a^	48.7%	50.1%	48.5%	45.3%
Talked to teacher/coach about college (Yes)^a^	49.7%	49.2%	55.0%	44.3%
*School Discipline and School Policing*				
Ever been suspended or expelled in past 2 years?	32.1%	40.9%	26.3%	16.0%
Police officer or officers regularly stationed at school?	81.8%	82.7%	81.5%	80.0%
Some other security guard regularly stationed at school?	71.5%	78.2%	71.0%	52.6%
Stopped by police at school	22.3%	20.9%	25.4%	23.3%
Ever seen someone stopped by police in your school?	50.5%	51.9%	49.4%	48.1%
*School Connectedness*				
I feel like I am part of my school (Yes)	90.6%	89.8%	91.4%	91.8%
I feel close to people at my school (Yes)	90.4%	88.3%	93.0%	92.6%
I am happy to be at my school (Yes)	89.8%	86.8%	93.8%	92.2%
I feel safe at my school (Yes)	93.7%	91.9%	95.0%	96.8%
Teachers in school treat the students with respect (Yes)	93.7%	91.0%	97.1%	96.1%

*Note*. Percentages based on valid cases for each variable.

a Conditional on having talked to an adult about college (n = 999); denominators are Black n = 547, Latino n = 260, White n = 192,

* p < .05;

** p < .01;

*** p < .001.

**Table 2.** Model Fit Indices for Latent Class Analysis (1–4 Classes)

Model	AIC	BIC	aBIC	Entropy	LMR-LRT (p)	BLRT (p)
1-Class	17789.71	17864.28	17819.81	—	—	—
2-Class	17660.01	17814.47	17722.35	0.57	<.0001	<.0001
3-Class	17544.85	17779.21	17639.44	0.67	<.0001	<.0001
4-Class	17466.16	17780.42	17593.00	0.67	.0001	<.0001

*Note*. AIC = Akaike Information Criterion; BIC = Bayesian Information Criterion; aBIC = Sample-Size Adjusted BIC; LMR-LRT = Lo-Mendell-Rubin Likelihood Ratio Test; BLRT = Parametric Bootstrapped Likelihood Ratio Test. The 4-class solution was selected based on the lowest BIC, significant BLRT, and substantive interpretability. Entropy values for the 3- and 4-class solutions were equivalent (0.67). AvePP for the four-class solution: Class 1 = 0.809, Class 2 = 0.787, Class 3 = 0.936, Class 4 = 0.840, all exceeding the 0.70 threshold for acceptable classification quality. The BLRT for the four-class solution was based on five successful bootstrap draws; the significant LMR-LRT (p = .0001) independently corroborates class selection.

**Table 3.** Conditional Probabilities for the Four-Class Solution

Indicator	Class 1 Surveilled but Supported (43.0%)	Class 2 Vicarious Policing (29.7%)	Class 3 The Criminalized (4.0%)	Class 4 The Protected (23.4%)
n	653	451	61	355
*College Aspirations*				
How important to you that you graduate from high school? (Important)	1.00	1.00	0.96	1.00
How important to you that you graduate from college? (Important)	1.00	1.00	0.57	1.00
How likely are you to graduate from college? (Likely)	1.00	1.00	0.51	0.98
*College-Going Conversations*				
Have you talked to an adult about applying to college?	0.56	1.00	0.30	0.59
Talked to parent/guardian about college	0.51	0.38	0.26	0.71
Talked to guidance counselor about college	0.15	1.00	0.39	0.23
Talked to teacher/coach about college	0.69	0.31	0.35	0.52
*School Discipline and School Policing*				
Ever been suspended or expelled in past 2 years?	0.45	0.28	0.52	0.13
Police officer or officers regularly stationed at school?	0.84	0.86	0.97	0.72
Some other security guard regularly stationed at school?	0.83	0.80	0.69	0.49
Stopped by police at school	0.23	0.22	0.38	0.16
Ever seen someone stopped by police in your school?	0.53	0.58	0.63	0.38
Race/Ethnicity				
Black	0.71	0.59	0.57	0.23
Latino	0.29	0.27	0.21	0.28
White	0.00	0.15	0.23	0.50

*Note*. Probabilities indicate the likelihood of a Yes or Important/Likely response for each indicator, conditional on class membership. Race/ethnicity probabilities indicate the proportion of each racial group within the class. Class 3 (n = 61) should be interpreted with caution given the small cell size.

**Table 4.** Multiply Imputed Auxiliary Multinomial Logistic Regression Predicting Class Membership (Reference: Class 4, “The Protected”; N = 1,520)

	Class 1 Surveilled but Supported	Class 2 Vicarious Policing	Class 3 The Criminalized
Covariates	*OR [95% CI]*	*OR [95% CI]*	*OR [95% CI]*
*Demographics*			
Maternal Marital Status: Married (Ref: Not Married)	0.055 [0.020, 0.152]*	0.255 [0.164, 0.398]*	0.260 [0.113, 0.597]*
Maternal Education: HS/Equivalent (Ref: < HS)	1.45 [0.78, 2.68]	1.39 [0.84, 2.32]	1.17 [0.52, 2.66]
Maternal Income: $15k-$34,999 (Ref: < $15k)	0.55 [0.22, 1.35]	0.89 [0.42, 1.85]	0.90 [0.29, 2.78]
Maternal Nativity: Born in U.S. (Ref: Foreign Born)	0.43 [0.13, 1.47]	1.13 [0.54, 2.36]	0.61 [0.16, 2.34]
*School-level Connectedness*			
I feel like I am part of my school	1.33 [0.49, 3.62]	2.61 [0.95, 7.15]	0.64 [0.21, 1.98]
I feel close to people at my school	0.81 [0.30, 2.17]	1.09 [0.44, 2.74]	0.31 [0.10, 0.95]
I am happy to be at my school	0.55 [0.22, 1.35]	0.70 [0.30, 1.65]	0.64 [0.21, 1.98]
I feel safe at my school	1.58 [0.66, 3.80]	1.46 [0.63, 3.38]	0.40 [0.07, 2.24]
Teachers in school treat the students with respect	0.75 [0.12, 4.57]	0.38 [0.10, 1.41]	0.60 [0.10, 3.49]

*Note*. OR = Odds Ratio; CI = Confidence Interval. Reference class = Class 4 (The Protected). Odds ratios derived from multiply imputed R3STEP auxiliary regression across 20 imputed datasets (N = 1,520).

* p < .05 (95% CI excludes 1.0). Results were substantively stable across listwise (n = 1,137) and multiply imputed specifications.

**Table 5.** BCHC Sensitivity Analysis: Equality Tests of Distal Categorical Outcomes Across Carcerality-Only Latent Classes

Distal Outcomes	Class 1 Yes % (OR [95% CI])	Class 2 Yes % (OR [95% CI])	Class 3 Yes % (OR [95% CI])	Class 4 Yes % (Reference)	Overall χ^2^	df	p
*College Aspirations*							
How important to you that you graduate from high school?	99.4% OR = 0.49 [0.03, 9.01]^c^	100.0% OR = n/e^a^	99.1% OR = 0.35 [0.01, 8.43]^c^	99.7% *(Reference)*	285.12	3	<.001
How important to you that you graduate from college?	98.5% OR = 1.79 [0.32, 9.93]	97.4% OR = 1.05 [0.28, 3.96]	97.2% OR = 0.97 [0.19, 4.82]	97.3% *(Reference)*	0.51	3	.917
How likely are you to graduate from college?	99.4% OR = 5.48 [0.10, 297.14]^c^	95.4% OR = 0.68 [0.21, 2.13]	94.1% OR = 0.52 [0.15, 1.82]	96.9% *(Reference)*	2.04	3	.564
*College-Going Conversations*							
Have you talked to an adult about applying to college?	71.5% OR = 1.75 [1.10, 2.78]	65.7% OR = 1.33 [0.85, 2.08]	68.3% OR = 1.50 [0.86, 2.63]	58.9% *(Reference)*	6.41	3	.093
Talked to parent/guardian about college^b^	54.4% OR = 0.99 [0.59, 1.67]	50.0% OR = 0.83 [0.47, 1.45]	41.1% OR = 0.58 [0.30, 1.13]	54.7% *(Reference)*	3.18	3	.365
Talked to guidance counselor about college^b^	52.6% OR = 1.50 [0.89, 2.53]	50.5% OR = 1.37 [0.78, 2.41]	45.6% OR = 1.13 [0.58, 2.19]	42.6% *(Reference)*	2.74	3	.434
Talked to teacher/coach about college^b^	58.3% OR = 1.67 [0.98, 2.83]	47.4% OR = 1.08 [0.61, 1.89]	46.3% OR = 1.03 [0.53, 1.98]	45.6% *(Reference)*	4.29	3	.232

*Note*. BCHC = categorical extension of the Bolck-Croon-Hagenaars correction procedure (Bolck et al., 2004; Vermunt, 2010; Asparouhov & Muthén, 2021), used here to test whether college aspiration and college-going behavior items differ across latent classes defined solely by carceral exposure (suspension, police officer stationed, security guard stationed, police stop, witnessing stops, race). “Yes %” is the proportion of respondents in each class who endorsed the item. Odds ratios (OR) compare the odds of an affirmative response in Classes 1 through 3 to the odds in Class 4, which serves as the reference class (OR = 1.00 by definition); an OR above 1.00 indicates higher odds of endorsement relative to Class 4, and an OR below 1.00 indicates lower odds. Brackets show the 95% confidence interval; when the interval crosses 1.00, the difference from the reference class is not statistically significant. BCH entropy (0.530) was substantially lower than the primary four-class solution entropy (0.666), which is expected because the BCH class solution was estimated using only carceral indicators (excluding the aspiration and behavior items), and further supports the primary specification. BCH class sizes: Class 1 = 24.1%, Class 2 = 40.3%, Class 3 = 11.9%, Class 4 = 23.8%.

a Class 2 answered this item affirmatively with near-total consistency (100.0%), producing a boundary probability estimate; the corresponding odds ratio and confidence interval could not be reliably estimated (n/e = not estimable) and are omitted rather than reported as spuriously large.

b Parent/Guardian, Guidance Counselor, and Teacher/Coach were asked only of respondents who indicated having talked to an adult about applying to college; respondents who answered no to that item skipped these three follow-up items by design. Results for these three outcomes are based on n = 999 and should be interpreted as comparisons among respondents who had already had some form of adult conversation about college, not as population-wide estimates.

c Several odds ratios for the College Aspirations items (e.g., HS Graduation Important, College Likely) have wide confidence intervals spanning less than 1 to well above 10. This reflects sparse cells: very few respondents in some classes answered no to these items, so the odds ratio comparing that small group to the reference class is estimated imprecisely. The corresponding overall chi-square tests and p-values, which do not depend on this instability, are the more reliable basis for judging whether classes differ; the odds ratios and intervals are reported for descriptive completeness but should be interpreted cautiously where cell sizes are small.
